# Transcriptome Analysis of *Spermophilus lateralis* and *Spermophilus tridecemlineatus* Liver Does Not Suggest the Presence of Spermophilus-Liver-Specific Reference Genes

**DOI:** 10.1155/2013/361321

**Published:** 2013-05-25

**Authors:** Bryan M. H. Keng, Oliver Y. W. Chan, Sean S. J. Heng, Maurice H. T. Ling

**Affiliations:** ^1^Raffles Institution, One Raffles Institution Lane, Singapore 575954; ^2^Department of Zoology, The University of Melbourne, Genetics Lane, Parkville, VIC 3010, Australia; ^3^Department of Mathematics and Statistics, South Dakota State University, SD 57007, USA

## Abstract

The expressions of reference genes used in gene expression studies are assumed to be stable under most circumstances. However, studies had demonstrated that genes assumed to be stably expressed in a species are not necessarily stably expressed in other organisms. This study aims to evaluate the likelihood of genus-specific reference genes for liver using comparable microarray datasets from *Spermophilus lateralis* and *Spermophilus tridecemlineatus*. The coefficient of variance (CV) of each probe was calculated and there were 178 probes common between the lowest 10% CV of both datasets (*n* = 1258). All 3 lists were analysed by NormFinder. Our results suggest that the most invariant probe for *S. tridecemlineatus* was 02n12, while that for *S. lateralis* was 24j21. However, our results showed that Probes 02n12 and 24j21 are ranked 8644 and 926 in terms of invariancy for *S. lateralis* and *S. tridecemlineatus* respectively. This suggests the lack of common liver-specific reference probes for both *S. lateralis* and *S. tridecemlineatus*. Given that *S. lateralis* and *S. tridecemlineatus* are closely related species and the datasets are comparable, our results do not support the presence of genus-specific reference genes.

## 1. Introduction

Gene expression analysis is examining the variations in gene expression by measuring DNA expression levels over time. These variations may be a result of many factors, such as environmental, developmental, and metabolic changes, or treatments. Quantitative real-time polymerase chain reaction (qRT-PCR) is one such used technique to quantify and analyse gene expressions [1, 2]. However, qRT-PCR requires a stably expressed gene under a wide variety of conditions [3, 4], known as a reference gene, as a standard to produce accurate and reliable results on transcriptional differences of various genes of interest. 

Candidate reference genes, which are commonly assumed to be invariant, can be identified using statistically based algorithms, such as geNorm [5], NormFinder [6], and BestKeeper [7], or descriptive statistics, such as regression [8]. Microarrays, which usually contain thousands of probes, present a good source of data for identifying reference genes [9]. Reference genes had been successfully identified from microarrays in a number of studies [10, 11].

However, several studies had refuted the possibility of universal reference genes [10–14] that can be used in every organ in every organism. This corroborates several studies demonstrating that genes commonly considered to be expressionally invariable may vary under different experimental conditions [15–17]. Some studies had verified the applicability of commonly used reference genes such as *GAPDH *(*glyceraldehyde-3-phosphate dehydrogenase*) [18] or *UBQ *(*ubiquinone*) [18]. However, other studies had demonstrated that the expressions of *GAPDH* [19] and *UBQ *[20] vary in some conditions. *Polr32 *has been suggested to be stably expressed in mouse heart [21], but Mamo et al. [22] had shown that *Polr32* is not stably expressed in mouse oocytes and embryos. *GAPDH* and *PPIA* are suitable reference genes for the human heart [23], but these genes were found to be unsuitable reference genes [24] for the human brain, dura mater, and meningiomas. These suggest that established reference genes for a particular organism may not be suitable for other organisms [25]. Using expressionally variable genes as reference genes will confound the results as it will be impossible to attribute the variation of the reference gene from the gene of interest. Given the lack of universal reference genes and the unlikelihood of organism-specific reference genes for multicellular organisms, the possibility of reference genes that are both organ specific and lineage-specific had been proposed [11]. In bacteria, lineage-specificity reference genes referred to suitable reference genes across different species, such as across genus (known as genus-specific reference genes) or across different families (known as family-specific reference genes). Hence, reference genes both lineage-specific and organ specific (known as lineage-organ specific reference genes) referred to suitable reference genes for a particular organ across different species. For example, a suitable spleen reference gene suitable for all species of mammals would be known as mammal-spleen-specific reference gene. However, if the reference gene was only suitable for marsupial spleen, then it would be known as a marsupial-spleen-specific reference gene.

In this study, we evaluated 2 liver transcriptome microarray datasets from *S. lateralis* and *S. tridecemlineatus* to draw conclusions as to whether there are reference genes that are both genus specific and liver specific. The liver transcriptomes of *Spermophilus lateralis, *a ground-roaming golden-mantle ground squirrel, and *Spermophilus tridecemlineatus*, a tree-habiting 13-lined ground squirrel, under different states of feeding and hibernation had been studied using the same microarray platform (GPL 1706; [26]). This allows for transcriptome comparison between two closely related species as the experimental conditions were largely similar.

## 2. Materials and Methods

### 2.1. Microarray Data

Two datasets were obtained from publicly available microarray database, Gene Expression Omnibus, National Centre for Biotechnology Information, of which one of them was from *S. lateralis *consisting of 35 samples (GSE2024) and another was from *S. tridecemlineatus* consisting of 26 samples (GSE2021). Briefly, these gene expression datasets represent the liver transcriptome for animals sampled during summer, interbout arousal, and late torpor [26].

### 2.2. Normalization across Data Sets

The intensity of each probe was calculated as the log-ratio of liver reference against sample. After which, each original probe log-ratio value in the original dataset was normalized using *Z*-score transformation based on the method previously described [11, 27]. Briefly, *Z*-score_Probe_ = (Probe_Initial_ × (*µ*_InitialProbe_/*µ*_Assumed_) − *µ*_Assumed_)/SD_Dataset_, where Probe_Initial_ is the original probe log-ratio intensity value in the dataset, *µ*_InitialProbe_ is the mean of the initial probe log-ratio values for a particular sample, *µ*_Assumed_ is mean of both datasets consisting of 61 samples, and SD_Dataset_ is the standard deviation for each initial dataset. The *Z-*scores for each probe across different original datasets will then be comparable. The absolute values for *Z-*scores were used for further analyses.

### 2.3. Determining Correlation between Datasets

**Figure 1:**
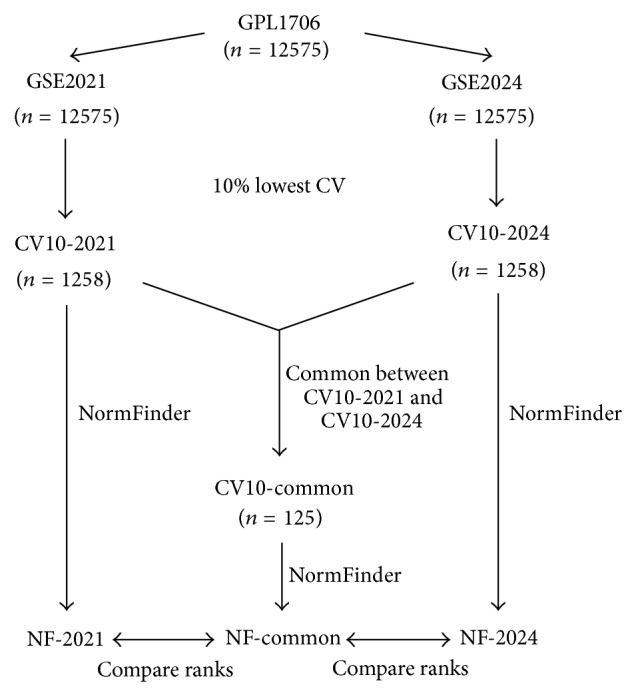
Summary of materials and methods. Two datasets, GSE2021 and GSE2024, were obtained from NCBI GEO, and probes were ranked by coefficient of variation (CV). Probes with the lowest decile in terms of CV were identified from each dataset as CV10-2021 and CV10-2014, respectively. The set of probes found in both CV10-2021 and CV10-2014 was identified as CV10-common. All 3 sets: CV10-2021, CV10-2014, and CV10-common, were analyzed by NormFinder [6].

The coefficient of variation (CV) of every probe was calculated as the quotient of standard deviation and arithmetic mean. From 12575 probes, the probes were ranked in ascending CV for each dataset, and the rank difference was calculated. Spearman's correlation was used to determine the correlation between the CV values between GSE2021 and GSE2024. From 12575 probes, the data was separated into groups of 10% each. The top 10% of probes in GSE2021 and GSE2024 were identified. These were called CV10-2021 and CV10-2024, respectively (Figure 1). A third list, CV10-common, was defined as common probes between CV10-2021 and CV10-2024. All three lists were analysed using NormFinder version 0.953 [6] to rank the stability of these probes separately by estimating the expressional variation of the probe with respect to the overall variation in the entire dataset. The resulting NormFinder outputs from CV10-2021, CV10-2024, and CV10-common were referred to as NF-2021, NF-2024, and NF-common, respectively. Spearman's correlation of the NormFinder stability index values between all sets was calculated and evaluated for the null hypothesis of no correlation between the data sets using *Z*-test for correlation coefficient [8, 28].

### 2.4. Bootstrap Statistics

The significance of overlap, CV10-common, was determined using bootstrapping method [29] where the percentage of overlap is the test statistic. The bootstrap distribution was generated by the percentage overlap of 2000 repetitive resampling from the original data with replacement to yield samples of the same sizes as that used to calculate test statistic (see Appendix A). *Z*-test was performed on the test statistic using the mean and standard deviation of the bootstrap distribution.

### 2.5. Expression Correlation of Invariant Probes

The expression correlations of the invariant probes with other probes in GSE2021 were found. This tests the hypothesis that the expressions of invariant probes were less correlated to the transcriptome compared to randomly selected probes. Ten least variant probes (10 most expressionally stable probes) were identified from NF-2021 and denoted as NF-10. Ten probes which were not in NF-10 were randomly selected from GSE2021 (*n* = 12565) and denoted as Random-10. The rest of the 12,555 probes that were not in NF-10 or Random-10 were denoted as Others-2021. Pair-wise Pearson's correlation is performed between each of the probes in NF-10 and each of the probes in Others-2021 to give a sample of the expression correlation of invariant probes and other probes (total  correlations = 125,550). The random correlations were estimated by pair-wise correlation between each probe in Random-10 and Others-2021. To prevent biasness, the process of randomly selecting 10 probes that were not in NF-10 and pair-wise correlation calculation was repeated 5 times.

## 3. Results

The two datasets used in this study, GSE2021 and GSE2024, were of the same microarray platform (GPL1706), examining differential gene expressions in the liver of 2 species of squirrels in similar states of hibernation and activity. This allows for a comparative study to evaluate potential genus-specific and organ-specific reference genes. The microarray consists of 12575 probes. Spearman's correlation coefficient of the coefficient of variation (CV) of the probes in GSE2021 and GSE2024 after *Z-*normalization was 0.141 (*P* value = 1*e* − 56), suggesting correlation between both datasets.

Among the lowest 10% of CV in both datasets (CV10-2021 and CV10-2024, *n* = 1258), 178 probes (14.2%) were common in both datasets, which is statistically significant from a bootstrap mean of 43.11% with a bootstrap standard deviation of 1.33% (*P*  value < 1*e* − 300) as resampled from the lowest 10% of CV in both datasets. However, Spearmans correlation for the NormFinder ranks of these 178 common probes between GSE2021 and GSE2024 was 0.322, and *Z*-test suggests that the ranks are correlated (*P*  value = 1*e* − 5). 

CV10-2021, CV10-2024, and CV10-common were analysed by NormFinder. There is one common probe (Probe ID 07I20, Table 1) between the 20 most invariant probes of NF-2021 and NFC-2021 (*S. tridecemlineatus*). NFC-2021 is the result of NormFinder analysis of CV10-common, using data from GSE2021. Coversely, NFC-2024 is the result of NormFinder analysis of CV10-common, using data from GSE2024. When ranked by NormFinder stability indices, the ranks of Probe ID 07I20 are 926 and 127 in NF-2024 and NFC-2024 (*S. lateralis*), respectively.

However, there are 6 common probes in the 20 most invariant probes of NF-2024 and NFC-2024 (*S. lateralis*; Table 2), including the top 2 invariant probes, 24j21 and 12c07. The NormFinder stability ranks of Probe IDs 24j21, 12c07, 24n06, 12g19, 15b23, and 12j13 in NF-2021 (*S. tridecemlineatus*) are 8644, 84, 321, 225, 654, and 650, respectively. Similarly, the NormFinder stability ranks of Probe IDs 24j21, 12c07, 24n06, 12g19, 15b23, and 12j13 in NFC-2021 (*S. tridecemlineatus*) are 127, 4, 53, 19, 101, and 89, respectively.

There is no common probe found within all 4 lists, namely, NF-2021, NFC-2021, NF-2024, and NFC-2024. 

**Figure 2:**
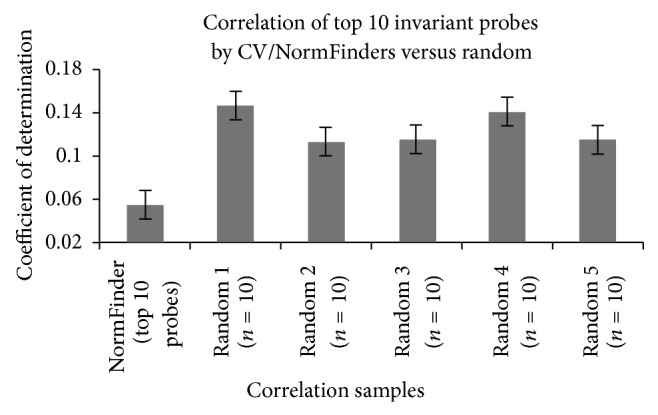
Average correlation of Top 10 invariant probes by CV/NormFinder (NF-10) against random (Random-10). Five replicates of correlations between Random-10 and Others-2021 were performed. Error bars denote standard error.

The average coefficient of determination (*r* ^2^) between 10 of the most invariant probes of GSE2021 (NF-10) and Others-2021 (those probes not found in NF-10 or Random-10) was 0.055 with standard deviation of 0.136. The average coefficient of determination (*r* ^2^) of the 5 replicates between (Random-10) and Others-2021 ranged from 0.115 to 0.147 with standard deviation ranged from 0.133 to 0.167 (Figure 2). Using *t*-test with unequal variance, the *r* ^2^ between NF-10 and Others-2021 was significantly lower than the *r* ^2^ between Random-2021 and Others-2021 (*P*  value < 1*e* − 300). By calculating Pearson's correlations of the expression of a probe with itself, the floating-point error in calculation can be estimated to be zero with a standard deviation of 2.79*e* − 12. Hence, we reject the null hypothesis that the correlations between invariant probes and random variant probes are equal and accept the alternate hypothesis that they are significantly lower.

## 4. Discussion

Reference genes play a crucial role as standards for normalizing expression values in quantitative gene expression experiments. Common reference genes such as *GAPDH* [19] and *UBQ* [18] can be used over a very wide range of organisms, but individual studies suggested that these genes are not optimal and there exist more reliably stable genes within each single species [10, 21]. Genes that are suitable as reference genes for one organism may not be suitable for another [25]. However, the possibility of lineage-organ-specific reference genes had been proposed [11]. Using 2 microarray datasets from squirrel liver of 2 species under the same genus obtained under comparable conditions [26], this study aims to identify and evaluate potential genus-specific and liver-specific reference genes suitable for use in both *S. lateralis* and *S. tridecemlineatus*, as well as to draw a general conclusion to the similarity of the general gene expression levels in both species. 

Spearman's correlation (*P*  value = 1.00 × 10^−56^) for the entire dataset (*n* = 12575) based on CV ranks showed correlation between GSE 2021 and GSE 2024, suggesting transcriptional similarities between the livers of *S. lateralis* and *S. tridecemlineatus*. This supports Williams et al. [26]. Their paper found consistency in gene regulation between squirrel species, relating to different biological classes of genes. Such correlation in our data is expected as the two squirrel species are from the same genus. In addition, the data was collected from the same organ with the same experimental conditions [26]. Thus, the expression profiles are assumed to be largely similar. Moreover, Spearman's correlation for the 178 probes in NF-common also revealed a high correlation (*P*  value = 1.10 × 10^−5^). This is also expected, due to the overall correlation of the whole dataset. In addition, Natale et al. [30] found similar gene expressions after brain trauma in mice and rats of different genera. Albert et al. [31] found less than 1% differences between gene expression of dogs and wolves and other domesticated and wild animals. These results support our hypothesis.

The ratio of overlap between the 10% lowest CV of both datasets (CV10-common) is 14.2%, which is much lesser than the expected 43.1% from bootstrapping. This result is significant (*P* value < 1*e* − 300), showing that there is little correlation between the invariance of a gene in *S. lateralis* and its invariance in *S. tridecemlineatus*, which suggests that genus-specific reference genes are unlikely. Moreover, our results demonstrated a lack of commonality between the top 20 probes in NF-2021 and NF-2024. This implies that the suitable liver reference probes identified by NormFinder for *S. tridecemlineatus* (GSE2021) are not suitable for *S. lateralis*. 

However, there are 6 probes in the top 20 of NF2024 that also fall in NFC-2024 (NF-common of GSE2024), but these probes are ranked poorly in NF2021 (*S. tridecemlineatus*) with 84 the best rank. This suggests that there are 83 more suitable reference probes for *S. tridecemlineatus* if Probe 12c07 is to be used as a reference probe across both species. In addition, our results showed that the most stable probe for *S. lateralis* (Probe 24j21) is ranked 8644 in *S. tridecemlineatus*. Similarly, the common probe for NF-2021 and NFC-2021 (*S. tridecemlineatus*), Probe 07l20, is ranked lowly for *S. lateralis* (ranked 926 and 127 respectively). This is supported by our findings that there are no common probes in the top 20 NormFinder identified reference probes of NF-2021 (*S. tridecemlineatus*) and NF-2024 (*S. lateralis*). A suitable reference probe for *S. lateralis *and *S. tridecemlineatus*, by extension, a genus-liver-specific reference probe for *S. lateralis *and *S. tridecemlineatus,* should have low ranks in both datasets. Taken together, these results show that the reference probes suitable for one species may not be suitable for other closely related species, which also suggests that genus-specific reference genes are unlikely.

A plausible explanation for the previous results is gene function degeneracy across different species. It has been suggested that redundant genetic networks may be present to maintain network robustness of cellular function [32–34]. This indicates that not all genes may be critical because certain functional overlaps allow interchanging of functions due to functional degeneracy. Furthermore, it has also been suggested that cells have some buffer in genetic expressions [35–37]. Thus, when expression of a particular gene is altered, such a buffering mechanism enables different genes to fulfil a similar function. This is a possible explanation why the transcriptome of invariant genes between the *S. lateralis* and *S. tridecemlineatus *has a poor overlap for the genes of lowest invariance while there is significant correlation for the datasets as a whole, indicating that a particular function is not performed by the same genes in both species. Yet a previously conducted study [26] has stated that the overall liver functions of the 2 squirrels are similar. Therefore, this corroborates the hypothesis that different genes can serve a similar function.

There is discrepancy in the overlap of the sets of differentially expressed genes in the two hibernating species, implying that the overlaps are not as significant as expected. In other words, the genes are not closely related. This may be attributed to difficulties in the statistical detection of the modest transcriptional changes between the summer and winter animals, changes that may be masked due to interindividual variation and the difference in the number of individuals analyzed for each species (26 samples in GSE2021 and 35 samples in GSE2024) [26]. However, interindividual variation and different numbers of animals studied by Williams et al. [26] are unlikely to be substantial enough to result in a lack of suitable reference probes between 2 closely related species of squirrels. A possible explanation to reconcile the observation that overall liver functions of the 2 squirrels is similar [26] and yet demonstrating the lack of suitable common reference probes may be a situation whereby the topology of the gene expressions between the 2 species are similar but individual gene expressions may vary. Moreno-Sánchez et al. [38] and Ling et al. [28] had demonstrated that if 2 genes are expressionally correlated, there is a strong chance that these 2 genes are functionally related. Further studies examining the coexpression networks from these 2 datasets may be able to elucidate the impact of the expressional variation of individual genes on the topology of expression. 

Our results demonstrate that the correlation between the NF-10 probes (invariant) and the Others-2021 (variant) is low (*r* ^2^ = 0.055), whereas the coefficient of determination (*r* ^2^) between the randomly selected probes (Random-10) and probes from Others-2021 ranges from 0.115–0.147. This result is significant (*P*  value < 1*e* − 300), rejecting the null hypothesis that correlation between invariant probes (NF-probes) is equal to correlation between variant (random) probes. This supports the use of correlation as a method to identify reference probes/genes, which had been previously reported [39]. 

The data used in this study is from these two closely related species under the same experimental conditions and from the same organ. However, our results suggests that reference probes found in one species may not be suitable in other as we are not able to conclusively determine suitable genus-specific reference genes even in such closely related species and for the same organ. Despite having similar organ transcriptomes [26], our results do not support the presence of highly stable genus-organ-specific reference genes or probes even though we found several candidates of potential reference probes for both species using a lowest denominator approach. However, this also suggests that the suitability of potential reference genes or probes across more diverse species will be questionable. Therefore, it can be extrapolated that reference genes that are stable over an even wider range of organisms across genus and families would be unlikely to exist. This shows that for accurate experimentation, new reference genes should be identified for each organism and for each organ individually.

## 5. Conclusion

In conclusion, our results do not suggest the presence of a liver-specific reference gene/probe by analysing transcriptome profiles of 2 closely related species under similar conditions despite evidence supporting previous studies on the similarity of the transcriptomes. Hence, this study does not support the presence of genus-organ-specific reference gene. Therefore, reference genes should be identified for each organism and for each organ individually.

## Figures and Tables

**Algorithm 1:**
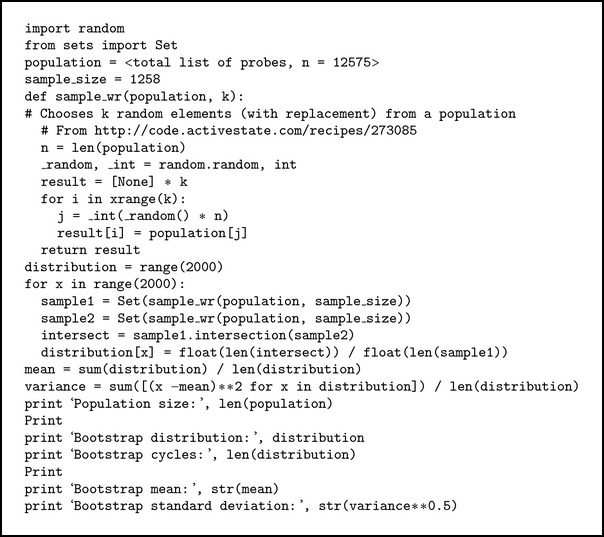
Python codes for overlap analysis by bootstrapping.

**Algorithm 2:**
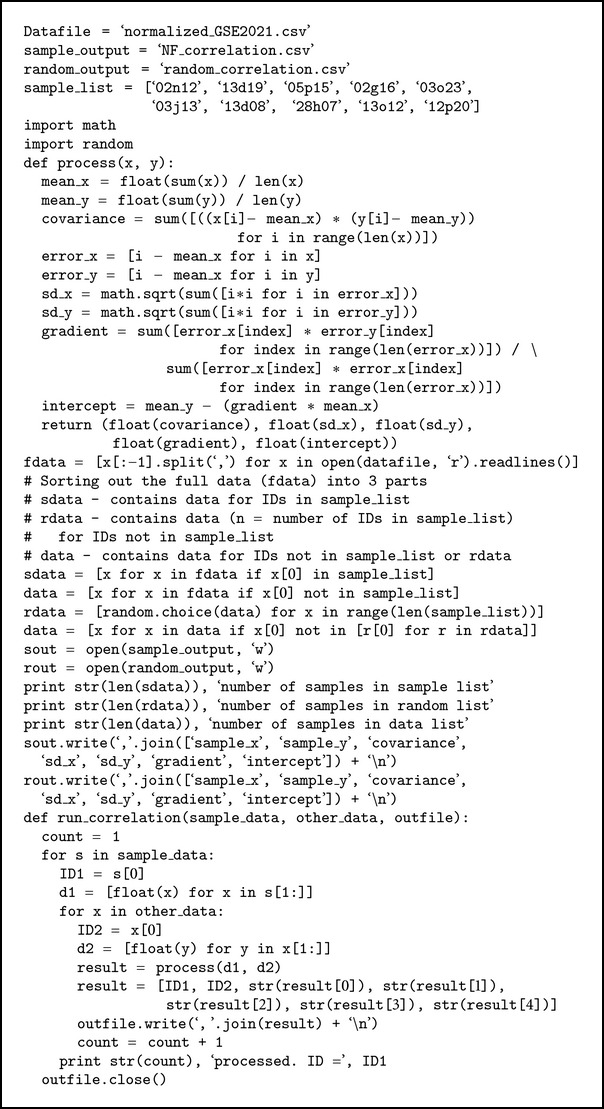
Python codes for expression analysis.

**Table 1:** Top 20 invariant probes of GSE2021 (*S. tridecemlineatus*) by NormFinder. NF-2021 is the result of NormFinder analysis of CV10-2021. NFC-2021 is the result of NormFinder analysis of CV10-common, using data from GSE2021. There is one common probe between NF-2024 and NFC-2024 as marked by asterisks.

NFC-2021		NF-2021	
Probe	Stability index	Probe	Stability index
12g21	0.230	02n12	0.167
07l20	0.230(∗)	13d19	0.195
01h05	0.245	05p15	0.197
12c07	0.247	02g16	0.201
01g24	0.259	03o23	0.206
15d12	0.267	03j13	0.208
12c08	0.272	13d08	0.208
01k05	0.281	28h07	0.219
12h15	0.282	13o12	0.220
01o21	0.282	12p20	0.221
12o20	0.291	28h10	0.225
23k15	0.291	28n23	0.229
15k15	0.292	26h02	0.233
12d19	0.295	13b20	0.237
12k13	0.296	07l20	0.243(∗)
23p12	0.299	15b12	0.246
23l15	0.319	13c20	0.249
26h04	0.322	01k09	0.251
12g19	0.323	03o04	0.251
28h04	0.328	12o11	0.252

**Table 2:** Top 20 invariant probes of GSE2024 (*S. lateralis*) by NormFinder. NF-2024 was the result of NormFinder analysis of CV10-2024. NFC-2024 was the result of NormFinder analysis of CV10-common, using data from GSE2024. Common probes in NF-2024 and NFC-2024 were marked by asterisks.

NFC-2024		NF-2024	
Probe	Stability index	Probe	Stability index
24j21	0.269(∗)	24j21	0.359(∗)
12c07	0.298(∗)	12c07	0.391(∗)
12g21	0.324	27j16	0.406
15k15	0.328	26b14	0.415
24n06	0.334(∗)	24n06	0.422(∗)
12g19	0.339(∗)	14p14	0.427
12j13	0.355(∗)	18o04	0.441
27o22	0.363	12g19	0.454(∗)
15b23	0.374(∗)	23n15	0.455
13k06	0.379	23n05	0.466
12c11	0.380	23h06	0.476
13k07	0.383	24h23	0.477
13j13	0.383	13l15	0.479
33p05	0.383	02o05	0.480
33p07	0.393	23n04	0.485
15g15	0.406	26g06	0.486
24n07	0.424	15b23	0.488(∗)
23j20	0.428	27c12	0.490
33e16	0.431	12f02	0.492
13b14	0.432	12j13	0.503(∗)

## Appendices

### A. Python Codes for Overlap Analysis by Bootstrapping

For more details, see Algorithm 1.

### B. Python Codes for Expression Analysis

For more details, see Algorithm 2.
